# Effect of the COVID-19 Pandemic on the Orthopaedic Surgery Residency Application Process: What Can We Learn?

**DOI:** 10.5435/JAAOSGlobal-D-21-00204

**Published:** 2021-10-04

**Authors:** Kevin Y. Wang, Jacob Babu, Bo Zhang, Meghana Jami, Farah Musharbash, Dawn LaPorte

**Affiliations:** From the Department of Orthopaedic Surgery, The Johns Hopkins University School of Medicine, Baltimore, MD.

## Abstract

The goal of this study was to assess the influence of the coronavirus disease 2019 pandemic on the orthopaedic surgery residency application process in the 2020 to 2021 application cycle.

**Methods:**

A survey was administered to the program directors of 152 Accreditation Council for Graduate Medical Education–accredited orthopaedic surgery residency programs. The following questions were assessed: virtual rotations, open houses/meet and greet events, social media, the selection criteria of applicants, the number of applications received by programs, and the number of interviews offered by programs.

**Results:**

Seventy-eight (51%) orthopaedic residency programs responded to the survey. Of those, 25 (32%) offered a virtual away rotation, and 57 (75%) held virtual open houses or meet and greet events. Thirteen of these programs (52%) reported virtual rotations as either “extremely important” or “very important.” A 355% increase was observed in social media utilization by residency programs between the 2019 to 2020 and 2020 to 2021 application cycles, with more programs finding social media to be “extremely helpful” or “very helpful” for recruiting applicants in 2020 to 2021 compared with the previous year (39% versus 10%, *P* < 0.001).

**Conclusion:**

Although many of the changes seen in the 2020 to 2021 application cycle were implemented by necessity, some of these changes were beneficial and may continue to be used in future application cycles.

Starting in mid-March 2019, the spread of the novel coronavirus disease 2019 (COVID-19) disrupted nearly every aspect of the US healthcare system, including graduate medical education. Owing to resource shortages and efforts to minimize medical student exposure to COVID-19, the Coalition for Physician Accountability enacted a variety of notable changes, including shortening or modifying clinical clerkships, eliminating away rotations, canceling US Medical Licensing Examination (USMLE), and transitioning the interview process to an entirely virtual format.^1^ During the 2020 to 2021 residency application cycle, 48,700 registered applicants were tasked to navigate the already rigorous endeavor of acquiring a residency position amid these unprecedented challenges.^2^

Specialties such as orthopaedic surgery which have historically placed much value on away rotations for evaluating applicants may have been particularly affected by the pandemic.^3-5^ Orthopaedic surgery is one of the most competitive specialties to match into, with a consistent average match rate from 2008 to 2018 of 77% and the average number of US applicants increasing by 15% over the past decade.^6^ Although the circumstances which led to the changes in the 2020 to 2021 application cycle were unprecedented, they provided a unique opportunity for residency programs to implement creative solutions and reevaluate the traditional residency application process. There have been recent reviews documenting the effect of the pandemic on orthopaedic resident education and case volume,^7-9^ but none to our knowledge has investigated the influence of the pandemic on the orthopaedic residency application process from the perspective of the residency program. Experiences from the pandemic may help to improve the application process for future cycles.

The goal of this study was to assess the influence of the COVID-19 pandemic on the orthopaedic surgery residency application process during the 2020 to 2021 application cycle compared with the previous year. We hypothesized that compared with the previous cycle, residency programs used social media substantially more to recruit applicants, placed greater importance on letters of recommendations (LOR) as well as standardized examination scores, and placed less weight on virtual rotations compared with away rotations. We further hypothesized that there was an increase in the number of applications received by programs and in the number of interviews offered by programs.

## Methods

### Survey Design and Administration

A survey was administered to all 152 Accreditation Council for Graduate Medical Education (ACGME)–accredited orthopaedic surgery residency programs using a web-based software (surveymonkey.com). Surveys were distributed to residency program directors or program coordinators, depending on the available contact information, with instructions for the residency program director to ultimately complete the survey. In total, 78 of the 152 ACGME-accredited orthopaedic surgery residency program directors (51.3% response rate) completed the survey. The survey contained 20 questions focusing on four key domains of the residency application cycle: virtual rotations, open house/meet and greet events, social media usage, as well as applications and resident selection. The survey was designed to assess changes in these domains from the 2019 to 2020 application cycle to the 2020 to 2021 cycle. Questions were phrased to elicit responses from the perspective of the residency program rather than from the perspective of the applicant. The questions included in the survey are detailed in Supplemental Table 1, http://links.lww.com/JG9/A163.

### Statistical Analysis

Descriptive statistics were done to analyze individual questions. To determine notable changes between the 2020 to 2021 application cycle and the previous year, we conducted bivariate statistics using chi-square tests for categorical variables, Student *t*-tests for parametric continuous variables, and Mann-Whitney *U*-tests for nonparametric continuous variables. The rank-choice questions assessing the importance of various factors in the applicant selection process were analyzed using an “average-ranking” algorithm based on the following equation: $\frac{x_{1}\;w_{1}+\;x_{2}\;w_{2}+\ldots +x_{n}\;w_{n}}{total\;response\;count}$, where *w* represents the weight of the ranked position and *x* represents the response count for each answer choice. This methodology has been previously validated for analyzing rank-choice survey questions.^10^ Furthermore, we subanalyzed our survey responses based on program size, which was defined as a binary variable (small versus large). Separating small and large residency programs based on the median residency size of all orthopaedic surgery residency programs, five residents per class, there were 41 small programs (≤5 Postgraduate Year 1 [PGY-1] residency positions) and 37 large programs (>5 PGY-1 residency positions) included in our analysis. Statistical significance was set at *P* < 0.05 for all statistical tests. All statistical analyses were done using STATA version 15.0 (StataCorp).

## Results

### Virtual Rotations

Of the programs which responded to the survey, 25 residency programs (32%) offered a virtual away rotation. Large programs were significantly more likely to offer virtual rotations than small programs (46% versus 20%, *P* = 0.012). The programs that did offer a virtual rotation had an average of 40.4 virtual rotators per program during the entire application cycle. Of all programs which responded, most programs viewed virtual rotations as “very important” (37%) or “somewhat important” (37%) to applicant selection for the 2020 to 2021 cycle, followed by “extremely important (16%),” “not so important” (5%), and “not at all important” (5%). Compared with in-person away rotations in the 2019 to 2020 cycle, virtual rotations were viewed as “extremely important” or “very important” by fewer programs (53% versus 88%, *P* < 0.001).

### Virtual Open House/Meet and Greet Events

During the 2020 to 2021 interview cycle, 59 programs (76%) that responded to our survey held virtual open houses or meet and greet events. Large programs were significantly more likely to offer virtual open houses or meet and greet events than small programs (89% versus 63%, *P* = 0.008). Most of these programs (79%) held meet and greet events “a few times” during the application cycle, compared with 17% who held them “at least monthly” and 4% who held them “at least weekly.”

### Social Media

Overall, more programs used social media for recruiting applicants in the 2020 to 2021 application cycle compared with the previous year (67% versus 15%, *P* < 0.001). Although a greater percentage of large programs used social media compared with small programs in the 2019 to 2020 application cycle (24% versus 5%, *P* = 0.014), this difference in social media utilization by program size was not statistically significant in the 2020 to 2021 cycle (70% versus 58%, *P* > 0.05). However, there still remained a greater percentage of large programs which used social medial compared with small programs when pooling the data across both years (47% versus 32%, *P* = 0.046). In addition, more programs found social media to be either “extremely helpful” or “very helpful” for recruiting applicants in the 2020 to 2021 cycle compared with the previous year (39% versus 10%, *P* < 0.001). Of the programs which used social media in the 2020 to 2021 application cycle, 88% used Instagram, 52% Twitter, 38% Facebook, and 4% LinkedIn.

### Applications and Resident Selection

**Figure 1:**
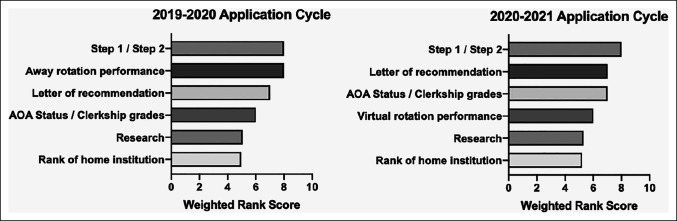
Graph showing comparison of the relative importance of various factors for applicant selection in the 2019 to 2020 versus 2020 to 2021 application cycles

During the 2019 to 2020 application cycle before the pandemic, the order of importance of factors for residency selection was (1) USMLE steps 1 and 2, (2) away rotation performance, (3) LOR, (4) Alpha Omega Alpha (AOA) honor society/clerkship performance, (5) research, and (6) rank of the applicant's home program (Figure 1). During the 2020 to 2021 application cycle occurring during the pandemic, the order of importance was (1) USMLE steps 1 and 2, (2) LOR, (3) AOA/clerkship performance, (4) virtual rotation performance, (5) research, and (6) rank of the applicant's home program (Figure 1). LOR and AOA/clerkship performance were ranked as more important in the 2020 to 2021 application cycle compared with the 2019 to 2020 cycle, whereas virtual rotations in the 2020 to 2021 application cycle were ranked as less important than away rotations in the 2019 to 2020 application cycle (*P* < 0.001 for all; Figure 1). These findings did not differ by program size (*P* > 0.05 for all).

Pooling data from both 2019 to 2020 and 2020 to 2021 application cycles, the average number of applications received by a program was 650 applications per year. Large programs received significantly more applications than small programs in both 2019 to 2020 application cycle (739 versus 558 applications, *P* < 0.001) and 2020 to 2021 application cycle (787 versus 558 applications (*P* < 0.001). The average number of interviews offered was 60 interviews per year and was higher for large programs than small programs in both 2019 to 2020 (75 versus 58 interviews, *P* = 0.012) and 2020 to 2021 application cycles (80 versus 56 interviews, *P* < 0.001). Across both years, the average number of interview dates was four per application cycle. The number of interview dates provided did not differ by program size in either application cycle (*P* > 0.05 for both). Furthermore, we did not detect a difference in the number of applications received per program, the number of interviews offered, or the number of interview dates offered between the two application cycles (*P* > 0.05 for all; Supplemental Table 1, http://links.lww.com/JG9/A163).

Most of the programs (89%) did not add supplemental requirements (for example, additional essays) for applicants in the 2020 to 2021 cycle. No difference was observed in the percentage of small versus large programs which had supplemental requirements (*P* > 0.05).

## Discussion

The disruption in the residency application process caused by the COVID-19 pandemic offered a unique opportunity for residency programs to implement novel strategies for promoting their programs and recruiting residency applicants. Although the temporary solutions implemented during the 2020 to 2021 application cycle were instituted by necessity, some of these changes may be applied in future residency application cycles as well. In this study, we surveyed ACGME-accredited orthopaedic surgery residency programs to examine how the pandemic influenced the residency application process in the 2020 to 2021 cycle compared with the previous year. Our key findings were as follows: (1) social media was used overwhelmingly more compared than in past years, with most programs using Instagram and Twitter, (2) virtual rotations were an important asset in the selection process, although less important than the away rotations in previous years, and (3) the total number of applications received and interviews offered per program did not change.

Our findings carry several important implications that may be used to improve the orthopaedic surgery residency application process in future application cycles. First, the pandemic seems to have further expedited an already progressing trend of residency programs using virtual platforms, such as social media, to recruit applicants. Based on our data from the survey respondents, there was a 355% increase in social media utilization by residency programs between the 2020 to 2021 application cycle and the previous year, with Instagram being the most popular platform and Twitter being the second most popular platform. This increase in social media utilization was driven by small programs, who saw an 11-times increase in social media utilization, compared with large programs whose utilization increased by only 2 times. During the pandemic, social media offered a distinct advantage in allowing residency programs to communicate information about their programs to applicants without in-person interactions. Furthermore, our data suggest that compared with large programs, small programs may have viewed social media as a more valuable tool for reaching out to potential applicants. Smaller programs, which may otherwise have less publicity than larger schools, may continue leveraging the reach of social media to advertise their program to more students in future application cycles.

Even before the 2020 to 2021 application cycle, residency programs had begun using the wide-reaching capabilities of social media. Some authors have noted that residency programs use social media to “brand” their programs.^11,12,13,14^ With the appealing visual content of Instagram and the “mention” and “hashtag” functions of Twitter allowing for seamless dissemination of information, social media generates an open dialogue between residency programs and applicants, which is important for conveying information during an application cycle. The pandemic seems to have accelerated the adaptation of social media into the residency application process, and it is likely that the niche role of social media in the application process will expand in future years. For programs without a social media presence, it may be helpful to begin cultivating this means of communication with their applicants, with a focus on Instagram and Twitter. From the applicant perspective, social media may become an increasingly reliable means of gathering nuanced information on residency programs that may not be readily available through more conventional avenues.

Second, although virtual rotations in the 2020 to 2021 cycle did not match the importance of away rotations in previous years, our data indicate that most programs still believed that virtual rotations were valuable for evaluating applicants. Historically, orthopaedic residency programs have relied heavily on away rotations for evaluating applicants, with over 50% of successful applicants matching at either their home program or a program they rotated at.^4,15^ A recent review found that, on average, an orthopaedic applicant completed 2.4 away rotations per application cycle.^16^ As a replacement for away rotations in the 2020 to 2021 application cycle, virtual rotations were launched at many residency programs to allow applicants to participate in resident educational activities, attend virtual social hours with residents, and even attend live-streamed surgeries.^17^

In a 2017 survey of 74 orthopaedic surgery program directors, O'Donnell et al^16^ found that both program directors and applicants perceived the value of away rotations as more utilitarian than educational. The authors reported that away rotations were most important for determining the “fit” of a program and for making a good impression at the program.^16^ Much of the subjective qualities of “fit” and of making a good impression are embedded in the in-person engagement inherent to away rotations, which cannot be replicated by virtual rotations. As a result, our study found that away rotations were the second most important factor for residency programs to evaluate applicants in the 2019 to 2020 cycle, compared with the 2020 to 2021 cycle where virtual rotations declined to the fourth most important factor. Notably, however, 53% of the programs responding to our survey still viewed virtual rotations as “very important” or “extremely important.” One downfall of away rotations is the cost because the average cost for a medical student to attend one orthopaedic away rotation in 2017 was estimated to be $2,799.^16^ In this aspect, virtual rotations likely provided cost savings to applicants during the 2020 to 2021 cycle. In addition, virtual rotations offer more flexibility which may allow applicants with scheduling conflicts to still engage with residency programs in meaningful ways. Although virtual rotations are unlikely to replace away rotations, there may still be a role for virtual rotations when COVID-19 restrictions are lifted, particularly for applicants who are unable to participate in an in-person rotation.

Third, there was increased importance placed on LOR and clerkship grades/AOA during the 2020 to 2021 application cycle relative to the previous year. In the 2019 to 2020 cycle, these two factors ranked below away rotations for importance. However, with the shift to virtual rotations in the 2020 to 2021 cycle, these two factors were ranked above virtual rotations. This finding may possibly mark a trend of clerkship performance becoming increasingly important for applicant selection. It is also possible that the transitioning of USMLE step 1, the most important selection factor across both application cycles, to pass/fail in 2022 may further elevate the relative importance of clerkship grades/AOA.^18^ To prepare for these changes, future applicants may benefit from increasing their focus on clerkship performance and forging meaningful relationships with surgeons who can provide strong letters of recommendation.

Finally, our analysis did not detect a change in the number of applications received by programs or the number of interviews offered to applicants in the 2020 to 2021 cycle compared with the previous year. We hypothesized that the convenience and cost savings of virtual interviews compared with in-person interviews would lead to an increase in the average number of applications received per program and the average number of interviews offered per program. Our data demonstrate this hypothesis to be false because we were not able to detect a difference in the number of applications received or interviews offered during the 2020 to 2021 application cycle compared with the previous year. Although several recent surveys have found virtual interviews to be a challenging way for applicants to learn about the culture of a program and obtain deeper insight into the training offered, virtual interviews do offer distinct advantages for convenience and eliminating travel costs, allowing applicants to schedule multiple interviews in a short span of time.^19,20^ In a recent survey of 1,711 medical students, Seifi et al^21^ reported that although most medical students prefer in-person interviews, most medical students also believe that virtual interviews should still remain an option for applicants. As such, although virtual interviews were a necessity during the pandemic, it is possible that some programs may still preserve the option of virtual interviews in future years.

The results of our study should be interpreted in the context of its limitations. First, there is a possibility of response bias among the programs who competed our survey, which may skew our results such that they are not generalizable to the entire national sample of orthopaedic residency programs. There are many reasons which may help explain why some programs did not respond to our survey—one reason being that the questions in the survey may have not been applicable to specific programs. However, our response rate of over 50% is notably higher than many previous survey studies administered to residency program directors.^22,23^ Second, because the surveys were administered after interview, invitations were released but before interviews were conducted, and we were unable to examine the perspective of the residency programs on the virtual interview process or on the match results. As such, it will be necessary for additional studies to comment on the opinion of orthopaedic surgery residency programs on virtual interviews and on the effect of COVID-19 on orthopaedic surgery match results. Third, because our survey was completed by residency program directors, we did not assess these data points from the perspective of the applicant. Finally, although we analyzed the effect of the pandemic on the volume of orthopaedic residency applications and interviews, we were not able to examine the phenomenon of “interview hoarding” by highly qualified applicants. Future investigations should analyze if the most highly qualified applicants received and attended a disproportionately greater number of interviews during the 2020 to 2021 application cycle compared with previous years. In addition, with orthopaedic surgery often cited being one of the least racially and sex diverse specialties in medicine, additional insight is needed concerning the influence of the pandemic on the diversity of applicants who applied, interviewed, and matched at orthopaedic residency programs.^24-26^

## Conclusion

The COVID-19 pandemic led to unprecedented challenges in the orthopaedic surgery residency application process. Our study surveyed ACGME-accredited orthopaedic surgery residency programs and found that, in the 2020 to 2021 application cycle, social media was used overwhelmingly more compared with the previous year and virtual rotations were viewed as important but less so than in-person rotations and that the number of applications received per program and interviews granted per program did not change compared with the previous year. Experience from the implementation of new ideas within the restraints imposed by the pandemic may be valuable in guiding the orthopaedic residency application process for future years.

## Supplementary Material

SUPPLEMENTARY MATERIAL
