# Supplementary material for: Effect of the COVID-19 Pandemic on the Orthopaedic Surgery Residency Application Process: What Can We Learn?
Source: J Am Acad Orthop Surg Glob Res Rev. 2021 Oct 4;5(10):e21.00204. doi: 10.5435/JAAOSGlobal-D-21-00204 (PMC8492373; doi:10.5435/JAAOSGlobal-D-21-00204)
Supplement: SUPPLEMENTARY MATERIAL [file jagrr-5-e21.00204-s001.docx]

**Supplemental Table 1. Survey Questions and Responses from Survey Administered to Orthopaedic Surgery Residency Program Directors**

| **Questions with Categorical Responses** | **Percentage of Program Directors** |
| --- | --- |
| **Did your program offer virtual rotations this year?** |  |
| *Yes* | 53% |
| *No* | 47% |
| **How important were virtual rotations for evaluating a candidate this year?** |  |
| *Extremely important* | 16% |
| *Very important* | 37% |
| *Somewhat important* | 37% |
| *Not so important* | 5% |
| *Not at all important* | 5% |
| **How important were in-person away rotations for evaluating a candidate last year?** |  |
| *Extremely important* | 54% |
| *Very important* | 33% |
| *Somewhat important* | 13% |
| *Not so important* | 0% |
| *Not at all important* | 0% |
| **Approximately how often did your program hold virtual open houses / meet & greets this year?** |  |
| *Never* | 25% |
| *A few times* | 59% |
| *At least monthly* | 13% |
| *At least weekly* | 3% |
| *More than weekly* | 0% |
| **Did your program utilize social media to reach out to applicants LAST YEAR?** |  |
| *Yes* | 15% |
| *No* | 85% |
| **Has your program utilized social media to reach out to applicants THIS YEAR?** |  |
| *Yes* | 67% |
| *No* | 33% |
| **If your program used social media this year, which platforms have you used? (Mark all that apply)** |  |
| *Twitter* | 52% |
| *Instagram* | 88% |
| *Facebook* | 38% |
| *Reddit* | 0% |
| *LinkedIn* | 4% |
| *Other* | CORD Google doc, Youtube Channel |
| **How helpful did you find social media to be for reaching out to applicants LAST YEAR?** |  |
| *Extremely helpful* | 2% |
| *Very helpful* | 8% |
| *Somewhat helpful* | 21% |
| *Not so helpful* | 21% |
| *Not at all helpful* | 48% |
| **How helpful do you find social media to be for reaching out to applicants THIS YEAR?** |  |
| *Extremely helpful* | 17% |
| *Very helpful* | 22% |
| *Somewhat helpful* | 39% |
| *Not so helpful* | 10% |
| *Not at all helpful* | 12% |
| **Has your program added supplemental requirements (additional essays, etc.) for applicants this year compared to previous years?** |  |
| *Yes* | 11% |
| *No* | 89% |
| **Questions with Quantitative Responses** | **Mean ± Standard Deviation** |
| *How many total students attended a virtual rotation at your program during the entire application cycle?* | 40 ± 31 |
| *How many applications did your program receive LAST YEAR?* | 630 ± 185 |
| *How many applications did your program receive THIS YEAR?* | 647 ± 213 |
| *How many applicants did your program offer interviews to LAST YEAR?* | 64 ± 27 |
| *How many applicants did your program offer interviews to THIS YEAR?* | 65 ± 24 |
| *How many interview dates were available LAST YEAR?* | 4 ± 7 |
| *How many interview dates were available THIS YEAR?* | 5 ± 6 |
